# Multidisciplinary Management of Radiation-Induced Salivary Gland Carcinomas in the Modern Radiotherapy Era

**DOI:** 10.3390/cancers12123769

**Published:** 2020-12-14

**Authors:** Domenico Attilio Romanello, Zulfiyya Imamguliyeva, Stefano Cavalieri, Barbara Vischioni, Lorenza Gandola, Alberto Iannalfi, Nicola Alessandro Iacovelli, Lisa Licitra, Marco Guzzo, Cesare Piazza, Davide Lombardi, Barbara Diletto, Pasquale Quattrone, Giuseppina Calareso, Laura Deborah Locati, Ester Orlandi

**Affiliations:** 1 Radiotherapy Unit, National Center of Oncological Hadrontherapy (CNAO), 27100 Pavia, Italy; domenico.romanello@cnao.it (D.A.R.); barbara.vischioni@cnao.it (B.V.); alberto.iannalfi@cnao.it (A.I.); ester.orlandi@cnao.it (E.O.); 2 School of Medicine, University of Milan-Bicocca, 20126 Milan, Italy; 3 Department of Head and Neck Tumors, National Center of Oncology, Baku AZ1012, Azerbaijan; dr.imamguliyeva@gmail.com; 4 Head and Neck Cancer Medical Oncology 3 Unit, Fondazione IRCCS Istituto Nazionale dei Tumori di Milano, 20133 Milan, Italy; lisa.licitra@unimi.it (L.L.); laura.locati@istitutotumori.mi.it (L.D.L.); 5 Radiotherapy 1 Unit, Fondazione IRCCS Istituto Nazionale dei Tumori, 20133 Milan, Italy; lorenza.gandola@istitutotumori.mi.it; 6 Radiotherapy 1-2 Unit, Fondazione IRCCS Istituto Nazionale dei Tumori, 20133 Milan, Italy; nicolaalessandro.iacovelli@istitutotumori.mi.it (N.A.I.); barbara.diletto@istitutotumori.mi.it (B.D.); 7 Department of Oncology and Hemato-Oncology, University of Milan, 20126 Milan, Italy; 8 Department of Otorhinolaryngology, Maxillofacial, and Thyroid Surgery, Fondazione IRCCS Istituto Nazionale dei Tumori, 20133 Milan, Italy; marco.guzzo@istitutotumori.mi.it; 9 Unit of Otorhinolaryngology-Head and Neck Surgery, Department of Medical and Surgical Specialties, Radiological Sciences, and Public Health, University of Brescia, 25123 Brescia, Italy; cesare.piazza@unibs.it (C.P.); davinter@libero.it (D.L.); 10 Pathology Department, Fondazione IRCCS Istituto Nazionale dei Tumori di Milano, 20133 Milan, Italy; pasquale.quattrone@istitutotumori.mi.it; 11 Department of Radiology, Fondazione IRCCS Istituto Nazionale dei Tumori di Milano, 20133 Milan, Italy; giuseppina.calareso@istitutotumori.mi.it

**Keywords:** Radiation induced cancer, salivary gland cancer, multidisciplinary management, radiotherapy, particle therapy, hormone therapy, surgery

## Abstract

**Simple Summary:**

Etiopathogenesis of salivary gland cancers [SGCs] is largely unknown, even if exposition to ionizing radiation is a recognized risk factor for SGCs development. To date, exhaustive data to guide clinicians in managing patients with radiation-induced [ri] SGCs are scarce and their treatment remains challenging. The purpose of this work is to describe and to analyze clinical and histopathological features, delivered treatments, and outcome of a series of patients with ri-SGCs treated at two Italian cancer referral sites. Given the rarity of ri-SGCs, this retrospective analysis conducted on a case series of 13 patients adds further knowledge to the paucity of literature. The management of these malignancies is extremely complex requiring a multidisciplinary treatment approach.

**Abstract:**

Clinical data of ri-SGCs patients treated between 2015 and 2019 at a tertiary cancer center and a national hadron therapy facility were reviewed. Latent time (LT) from first RT to ri-SGCs diagnosis, overall (OS), and disease-free survival (DFS) were assessed. Thirteen patients developed 14 ri-SGCs (one patient had 2 synchronous ri-SCGs), after a median LT of 23 years (range 16–34). Parotid was the primary site in 8 cases (57%) and salivary duct carcinoma was the most frequent histotype (29%). Nine patients (69%) underwent surgery (Sx). Among them, 4 patients (31%) underwent Sx alone, 5 received post-operative treatments: 3 (23%) photon-based (X) reRT, one (8%) protons and carbon ions, one (8%) carbon ions only. One patient (8%) received definitive XRT. The remaining 3 patients (23%) received androgen deprivation therapy. With a median follow-up of 48 months (range 24–72), median OS and PFS were 74 and 24 months, respectively. In the subgroup of AR^+^ ri-SGCs, median PFS and OS were 12 and 74 months, respectively. Given the rarity of ri-SGCs, this work adds further knowledge to the paucity of literature. The management of these malignancies is extremely complex requiring a multidisciplinary treatment approach.

## 1. Introduction

Malignant tumors of salivary glands (SGCs) comprise less than 0.5% of all cancers and constitute about 2–8.5% of head and neck cancers (HNCs) [1]. Worldwide annual incidence ranges from 0.4 to 2.6 cases over 100,000 [2] with a male to female ratio of 1.5:1, a higher prevalence in males compared to females during the 6th–7th decade for the salivary duct carcinoma (SDC), being exceedingly uncommon in children [1,2,3].

Etiopathogenesis of SGCs is largely unknown, even if exposition to ionizing radiation is a recognized risk factor for SGCs development. This was reported for the first time in Japanese survivors of the atomic bomb: the frequency of SGCs, mucoepidermoid carinoma (MEC) in particular, was disproportionately high at high radiation doses. In Japanese survivors, out of 145 SGCs registered from 1950 to 1987, 41 were malignant and the proportion of MEC raised with the increasing of radiation dose (*p* = 0.004 for linear trend) [4]. However, even doses of 2 Gy could raise the risk of developing salivary gland tumors [4,5].

Further evidence supporting the role of radiation in salivary gland carcinogenesis derives from data of children treated with scalp irradiation for tinea capitis, or who received radiotherapy (RT) to the head and neck area to reduce the size of the tonsils and adenoids [6,7]. These children had a 4.5-fold incidence of SGCs compared to untreated people with a mean latency period until tumor development of 11 years [6].

Globally, the standardized incidence ratio of HNCs in survivors of childhood cancer is 13.6 and SGCs represent 65% of these cases [8]. The risk has been associated with the primary tumor type, gender, type of chemotherapy, etc. Radiation has been associated with an increased risk of all carcinomas, most marked for HNCs (SIR 18.5), and the site of the second tumor had arisen in a previous radiation field in 85% of HNCs. Besides, a large study on children survivors after RT (with or without chemotherapy) for a first cancer, showed a linear correlation between dose exposure and the risk of developing solid tumors including SGCs, with the second malignancy occurring at least eight years after the end of the first oncological treatment [9].

Exhaustive data to guide clinicians in managing patients with ri-SGCs are scarce and their treatment remains challenging, especially in the era of targeted systemic treatments and innovative radiation therapies, namely intensity modulated RT (IMRT) and hadron therapy [9,10,11,12,13].

Ideally, surgery with wide margins remains the mainstay of treatment followed by RT in high-risk cases [14,15]. However, radical salvage surgery is often not feasible, essentially because of technical difficulties when operating within an irradiated area [9,12,16] or due to the local extension of disease that prevents a radical resection.

In addition, further RT could be hardly given since normal tissues surrounding secondary SGCs have been generally treated to near their dose tolerance. Finally, in some cases, patients who suffer from secondary SGCs are young and potentially candidates to develop a third cancer, in particular in the presence of a genetic susceptibility [17]. In this scenario, modern RT techniques, including IMRT and particle therapy, may play a principal role in the management of these patients. Indeed, thanks to their physical properties, they have an excellent sparing of normal tissue outside the target and they are able to overcome an enhanced hypo-oxygenation that can also be present in ri-SGCs [14].

With the limitations of the descriptive nature of our work and the limited patient cohort, the purpose of our review is to describe and to analyze clinical and histopathological features, delivered treatments, and outcome of a series of patients with ri-SGCs.

## 2. Results

### 2.1. Patients’ Characteristics

Patients’ clinical characteristics at the first cancer diagnosis are listed in Table 1.

Overall, we have identified 13 patients suitable for this analysis. One of these patients had two synchronous secondary SGCs after a LT of 34 years, for a total of 14 ri-SGC cases. Median age at diagnosis of primary tumor was 17 years (range 3–68), whereas median age at diagnosis of ri-SGC was 50 years (range 17–78), with a median interval between radiation exposure and diagnosis of the SGCs of 23 years (95% CI: 16–34).

Characteristics of the second SGCs were resumed in Table 2. Histology was quite heterogeneous: low grade MEC (*n* = 2), SDC (*n* = 4), low-grade polymorphous adenocarcinoma (*n* = 1), adenocarcinoma not otherwise specified NOS) (*n* = 3), carcinoma ex- pleomorphic adenoma (*n* = 1), adenoid-cystic carcinoma (ACC) (*n* = 2), basal cell adenocarcinoma (*n* = 1). Eight tumors occurred in the parotid glands, five in submandibular glands, one in minor salivary glands of the oropharynx. Positive nodes (*n* = 3) have been detected in submandibular (*n* = 2) or minor salivary glands (*n* = 1) tumors whereas distant metastases were present in 2 cases. Median LT to ri-SGCs was not significantly different whether primary tumor was diagnosed in pediatric age (31 years, range 5–50) or in adult (33.5 years, range 18–68), even though in the former diagnosis of ri-SGCS appeared earlier after primary treatment compared to the latter.

Immunohistochemical (IHC) research of androgen receptor has been done only in cases with diagnosis of SDC, adenocarcinoma NOS, and carcinoma ex pleomorphic adenoma. Seven out of 8 patients had androgen receptors (ARs) overexpression. HER2 overexpression was not identified. NGS was performed in 6 out of 13 (46%) cases, in 3 cases somatic tumor mutations have been found: BRCA2 mutation in one SDC, TP53 mutation in one adenocarcinoma NOS, PIK3CA and HRAS mutations in another SDC.

Concerning the relationship with the previous treated volumes, data were available for 9 (64%) ri-SGCs (one patient had two synchronous ri-SGCs, (Table 1). Six ri-SGCs developed within the previously treated volumes and in 5 of them, ri-SGC was marginal to high-dose volumes. Three ri-SGCs occurred within the initial target volume.

### 2.2. Treatment and Outcome for Secondary SGCs

Nine patients (69%) underwent surgery, either alone (*n* = 3), in combination with RT (*n* = 5), or with postoperative systemic treatment (*n* = 1); resection margins were positive in 8 cases. One patient received definitive RT. The remaining 3 patients received palliative treatment.

Seven patients (54%) out of 13 received RT as part of their multimodality treatment. Post-operative photon-based RT (XRT) (*n* = 3) doses ranged from 64 Gy to 70 Gy (median of 67 Gy); one patient received a palliative course of 30 Gy for an unresectable parotid gland tumor. Particle therapy was delivered in two cases: one patient was treated by post-operative Proton Therapy (PT) with a boost of Carbon Ion Radiotherapy (CIRT), receiving 6 Gy(RBE) with CIRT and 59.4 Gy with PT. The other one was treated with post-operative CIRT after microscopic residual surgery, with a total dose of 68.8 Gy(RBE). In the subgroup treated with post-operative RT, median age at primary tumor diagnosis was 12 years (range 3–27) and median LT was 17 years (range 5–41 years). The patient treated with radical RT was 8 years old at the time of the diagnosis of the primary tumor, with an LT of 37 years. With regard to acute toxicities, all of the patients who underwent post-operative RT developed mild to moderate xerostomia and oral mucositis (≤G2); as regards late toxicities, three patients treated with surgery and XRT developed buccal spasms (*n* = 1), fibrosis (*n* = 1), trismus and neuropathy of the facial nerve (*n* = 2). Both the 2 patients treated with post-operative particle therapy developed G1 trismus, G1 xerostomia, and G1 neuropathy of the facial and trigeminal nerve.

Cut-off date was October 2019.

Median follow-up was 48 months (95% CI: 24–72). Median OS was 74 months (95 % CI: 36.00–74.00). Median PFS was 24 months (95%CI: 6.00-upper limit not reached). Five-year OS and DFS were 67% and 26%, respectively. For the subgroup of AR^+^ SGCs, median DFS and OS were 12 months (95% CI: 2.00–25.00) and 74 months (95% CI: 13.00–74.00), respectively. In AR^−^ SGC patients, median DFS and OS were not reached; 2-year DFS was 62.5% and 2-year OS 100%.

## 3. Discussion

We reported a small series of 13 patients with ri-SGCs with a more favorable outcome than previously published older series. Median PFS and OS were 24 months (95% CI: 6 months-not reached) and 74 months (95% CI: 36–74), respectively, despite high-grade histology in 77% of cases and advanced stage (III–IV) in 10 out 13 cases. These results are very encouraging, if we consider that 9 patients (69%) received surgery, 1 patient (8%) was treated with definitive XRT and concomitant androgen deprivation therapy (ADT), 1 patient (8%) received palliative XRT and subsequent ADT, whereas the remaining 2 patients (15%) received only systemic therapy. In the past, a 5-year OS higher than 90% was reported, however, mucoepidermoid was the most common histotype and almost all patients received surgery combined or not with RT [9,12]. In the analysis by Bhattacharyya et al., for example, patients with MECs exhibited a higher 5-year OS (81.5%), compared to other histotypes such as ACC (70.7%) and carcinoma ex pleomorphic adenoma (40.2%) [18]. On the other hand, Mallik et al. reported 47 submandibular gland cancers, mostly ACCs, with a 5-year DFS of 71.8%, 12.8% (*n* = 6) of loco-regional failure, and 12.8% (*n* = 6) of development of distant metastases. Compared to the past, availability of new effective systemic treatments (e.g., androgen deprivation therapy, ADT; anti-HER2 monoclonal antibody), improvement in the re-irradiation (reRT) [19] techniques and the opening of heavy ions facility in Italy in 2011 have offered new therapeutic opportunities.

If MEC was the most common histotype [3,4,5,6,7,9,11,12,13,16,20,21,22,23,24,25] in previous series, the SGC histotypes reported in our series are quite heterogeneous: 9 out 13 (69%) were high-grade with AR expression in 7 out of 9 cases. Of note, 38 % received ADT with a median PFS and OS of 12 and 74 months, respectively. Activity of androgen blockade in AR expressing SGCs is already well known. Several papers [26,27,28] support the use of ADT in clinical practice and one randomized, prospective trial, is currently ongoing (NCT01969578). New potential druggable targets as HER2 [29], TRK [30], RET [31], BRAF [32] have been identified almost exclusively in high-risk histologies different from ACC, paving the way to active treatment approaches. Impressive results have been reported in the clinical practice with some tailored agents as trastuzumab emtansine [33], NTRK inhibitors [34,35], or RET inhibitors [36]. Activity of some antiangiogenic agents (e.g., lenvatinib) seems to be promising in ACC [37] even if structural disease shrinkage has been observed only in a few cases. Employ of checkpoint blockade (e.g., pembrolizumab) has been explored with a weak activity (ORR 12%) even if objective responses have been observed only in high-risk histotypes [38].

ReRT should be usually proposed as post-operative treatment, since it is related to prolonged survival rates, especially for ACC [39]: in our cohort, 5 patients out of 13 (38%) received a post-operative RT [any energy]. Indications for post-operative RT include advanced stage (T3, T4, *n*+), positive surgical margins, high tumor grade, perineural invasion, recurrent diseases [40,41]. Moreover, reRT should be taken into account whenever surgery is not feasible, even though no data from prospective randomized data are available in the literature. Nowadays, state-of-the-art RT techniques, such as IMRT and stereotactic body RT (SBRT) allow steep dose gradients with optimal coverage even for complex target volumes, while allowing a good spare of close healthy tissues. Following NCCN recommendations, reRT should take place at least 6 months after the end of the first RT course and target volumes should be taken as narrower as possible, avoiding elective nodes coverage. Results are encouraging: Karam et al. re-treated 18 consecutive patients with SBRT with a median dose of 30 Gy delivered in 5 fractions. With a median cumulative dose of 91.1 Gy, 2-years local control and OS were 53% and 39%, respectively, even if 4 patients eventually experienced soft tissue necrosis as a late severe toxicity [42].

In regards to IMRT, most of the experience in literature refers to mixed series where the number of SGCs is significantly lower than pharyngeal or laryngeal squamous cell carcinoma cases [19,43,44]. Recently, Orlandi et al. analyzed reRT outcomes on a cohort of 159 patients, of which 71 (45%) were not squamous cell carcinomas (in particular, 25 (16%) were ACC and 8 (5%) were affected by SGCs). With a median follow up of 49.9 months, 5-years OS and PFS were 43.5% (95% CI, 34.6–54.8%) and 20.9% (95% CI, 14.7–29.6%), respectively. It is hard to make comparisons with our current results, due to the limited number of patients and the mixed cohort of the cited study. To our knowledge, there are no data available in literature concerning reRT on an exclusive ri-SGCs cohort.

Treatment with hadrontherapy represents a new approach considering that most of these tumors arose in a previously operated and irradiated field. PT and CIRT seem to be a reasonable choice to overcome dosimetric thresholds in unresectable patients. Two patients (one teenager) received PT and CIRT (delivered doses: PT 59.4 Gy(RBE) + CIRT 6 Gy(RBE); CIRT 68.8 Gy(RBE)). The physical properties of PT can be exploited in pediatric patients. Through PT nearly the same physical dose of XRT can be delivered, with a neat fall of dose outside the target: Grant et al. reported a 53% of grade 2^−^ grade 3 (G2-G3) dermatitis, 0% of G2-G3 dysphagia, and a 46% of G2-G3 mucositis in a cohort of 13 pediatric patients receiving 60 Gy(RBE), compared to 11 patients treated with XRT (60 Gy, 54%, 27% and 91% of toxicities rates, respectively). Takagi et al. compared PT and CIRT in a cohort of 80 pts treated for head and neck ACC: no differences were found in terms of local control (66% for PT, 68% for CIRT) for T4 unresectable cases when a dose of 65.0 Gy(RBE) was delivered. CIRT combines the ballistic properties and superior biological equivalent dose, allowing to retreat unresectable radio-resistant tumors. With a median CIRT dose of 51 Gy(RBE) (27 × 3 Gy(RBE)) delivered 61 months after the first radiotherapy course, Jensen et al. reported an objective response rate of 57% and a local control (median follow-up 14 months) of 70%; in that cohort, median cumulative dose was 128 Gy(RBE), with acceptable toxicity rates [45,46]. The Italian experience concerning reRT of SGCs with CIRT has been recently published [47]: from 2013 to 2016, 51 patients were treated with a median dose of 60 Gy(RBE) at 3–5 Gy(RBE) per fraction. After a median follow-up of 19 months, the local control rate was 41.2%; one-year OS and PFS were 90.2% and 71.7%, respectively.

In line with other papers, the treatment was well tolerated, with no G4 or G5 toxicities and only 17.5% of G3 events. Moreover, compared to other series with CIRT [46,48], reRT at CNAO did not suffer from soft tissue necrosis or carotid blow-out syndrome events [47]. This was probably related to the lower biologically equivalent doses (BED) delivered—155.2–167.4 Gy(RBE)—which could likely be increased in order to improve the outcomes. Interestingly, Vischioni et al. reported a shorter median LT, 6.33 years (range 1.08–20) compared to our results.

Despite the clinical challenges facing by any HNC specialist (i.e., radiation oncologist, head and neck surgeons, oro-maxillofacial surgeons, medical oncologist) Gs, a multidisciplinary approach is needed when dealing with ri-SC. This approach has been strongly recommended to achieve the best oncologic outcome and prevent or adequately treat any adverse effects [49,50,51]. Indeed, it should be considered as mandatory for SGCs as well [52]. In our series, most patients did not receive radical surgery; this is not an unexpected finding since post-treatment fibrosis and distortion of anatomy due to the treatment[s] for the previous tumor may increase the rate of positive margins. In this scenario, post-operative RT can almost double local control rate [53], and our results confirm that among 5 patients receiving marginal Sx and post-operative RT, 4 (80%) had no evidence of disease at last follow up; the remaining one did, but he actually received CIRT. Data on the radiobiological response to this type of RT are still being collected, and a stable disease does not always translate into treatment failure [54]. Nevertheless, all of our patients treated by Sx and post-operative RT were alive at last follow up. In this context, a dedicated pathologist is advisable as SGCs show the greater discrepancy between the initial and definitive pathological diagnosis [55]; moreover, pathology must guide systemic therapy by identifying druggable targets. This approach allowed almost half of our patients to benefit from ADT, warranting at least 12 months of survival even without a curative treatment. Lastly, a multidisciplinary approach allows late toxicities to be more promptly identified and managed; this could explain the low number of severe sequelae among our patients and could result in a better quality of life.

RT is a well-known causative agent in the development of secondary solid tumors and the issue of second cancers following therapeutic radiation for a wide variety of malignancies is currently receiving increasing attention. Indeed, it is well recognized that patients receiving radiation therapy have a higher long-term risk for developing second primary cancers compared with patients who do not [13].

In pediatric patients, who constitute most of the cases originally treated in this study, germline mutations and hereditary conditions may place one at risk for developing a secondary malignancy, and may not be related solely to chemotherapy [25]. Considering that 10 patients in our series received radiation and chemotherapy compared to 3 patients who received radiation alone, our series suggests that the association of chemo-radiation may increase the risk of second SGCs compared to radiation alone. This may lead to the idea of treatment-induced second SGCs, rather than simply radio-induced secondary malignancies.

The main limitations of our research are its descriptive nature and the limited patient cohort. Beal KB et al. reported 18 radiation-induced salivary gland tumors, 15 of which were ri-SGCs [11]. The main strengths of this work are central pathologic review of the 12 non-ACC tumor specimens, the prolonged follow-up, and the details about treatments for ri-SGCs.

## 4. Materials and Methods

We retrospectively analyzed clinical data of consecutive patients with ri-SGCs referring to Fondazione IRCCS Istituto Nazionale dei Tumori, Milan, Italy, and to Centro Nazionale di Adroterapia Oncologica (CNAO, National Center of Oncological Hadrontherapy), Pavia, Italy, between 2015 and 2019. Patients were managed by head and neck and pediatric multidisciplinary teams established at both the former Institutions and involving dedicated radiation oncologists. We considered as eligible adult and pediatric patients with a confirmed pathological diagnosis of SGC, who received for previous tumors curative RT in HN areas, lung, and upper mediastinum, with or without concurrent or sequential chemotherapy. Multidisciplinary discussion has been considered within the inclusion criteria as well. Patients with primary leukemia, benign lesions, and patients who received only chemotherapy as a curative treatment have been excluded.

Staging procedures included HN computed tomography (CT) and/or magnetic resonance imaging (MRI), thorax and abdomen CT scan, or 18-FluoroDeoxyGlucose (FDG) Positron Emission Tomography (PET) total body scan.

Patients’ characteristics, first tumor diagnosis, and secondary SGCs features, latent time (LT) from initial treatment to the development of SGCs, treatment, and outcome for secondary SGCs were analyzed.

Revision of all specimens was performed by a dedicated HN pathologist (PQ). Immunohistochemical (IHC) profile including androgen receptors and HER2 has been done in SDCs and adenocarcinoma not otherwise specified (NOS). Next-generation sequencing (NGS) has been performed on the tumor specimen until it was available for free (September 2019). RT plan of the primary tumor, when possible, was reconstructed on CT planning of the secondary SGCs with the aim of searching for a relationship between the dose distribution of the first RT and the second malignant tumor. Median overall survival (OS) and progression-free survival (PFS) and their 95% confidence interval (CI) were estimated using the Kaplan–Meier method. Further subgroup analyses were realized according to histological features of SGCs.

This study was approved on June 2020 by the Ethical Committee of Fondazione IRCCS Istituto Nazionale dei Tumori, Milan, Italy (internal study identification number: INT 127-20).

## 5. Conclusions

Our findings underline the benefit of a multidisciplinary approach and intraspeciality care for the management of this complex clinical scenario. This is realized through tailored treatments which are properly balanced between tumor control and toxicity, as to allow similar outcome to ex novo SGCs.

## Figures and Tables

**Table 1:** Patient characteristics at diagnosis of primary cancer.

Patient	Gender	Age at Primary Diagnosis	Year of First Diagnosis	Histology	Site and Stage	Treatment	RT Techniques/RT Volumes and Doses	Late Sequelae	Latent Time between Primary Tumor and SGCs
Patient 1	F	10	2000	Medulloblastoma	Cerebellar vermis T3M0 (Chang system) High Risk (>1.5 cm^2^ residual disease)	Sequential high dose CT → Sx → > RT Vincristine and CCNU-based maintenance therapy	Conventional XRT Hyperfractionated accelerated RT (HART) Craniospinal axis: 39 Gy Posterior fossa: 60 Gy	Neurocognitive impairment Panhypopituitarism Bilateral hypoacusia	16 years
Patient 2	M	68	2008	Undifferentiated carcinoma EBER-negative HPV-positive	Nasopharynx cT2N2c (AJCC VII ed)	Platinum-based CRT	IMRT Elective nodal volume: 50 Gy High risk volume: 70 Gy Conventional fractionation	Severe Xerostomia Dental alterations Moderate fibrosis	10 years
Patient 3	M	18	1985	Nodular lymphocyte-predominant HL	Neck Stage IIA (Ann Arbor staging)	Neck Sx → RT	Conventional XRT: 40 Gy mantle field 45 Gy neck nodes	Chronic ischemic cardiomyopathy	32 years
Patient 4	M	40	1992	Squamous cell carcinoma	Oropharynx	Tonsillectomy → RT	Conventional XRT Elective nodal volume: 45 Gy High risk volume: 60 Gy Conventional fractionation	Xerostomia Hypothyroidism	23 years
Patient 5	F	27	1975	Papillary thyroid carcinoma	Thyroid (stage not available)	Total thyroidectomy> external beam RT	Conventional γ photon-based RT (Co60) 45 Gy	Xerostomia Moderate fibrosis	41 years
Patient 6	M	10	1997	Sarcoma G3, NOS	Soft tissues of paramandibular area (locally advanced disease, stage not available)	Sx → CT(CEVAIE) → RT(44.8 Gy, 1.6 Gy twice a day)	Conventional XRT	Moderate fibrosis	19 years
Patient 7	M	8	1975	HL	Neck Stage II (Ann Arbor staging)	CT→ RT	Conventional XRT	Moderate fibrosis	37 years
Patient 8	F	17	1985	Undifferentiated Carcinoma (EBER unknown)	Nasopharynx (loco-regionally advanced disease, stage not available)	CT (Vincristine, Adriamycin and cyclophosphamide × 2 cycles) RT + concurrent adriamycin and cyclophosphamide × 4 cycles	Conventional XRT T and pathological nodes: 62 Gy Elective nodes: 40.2 Gy	Trisma Xerostomia Massive fibrosis ipoplasia mandibola	34 years
Patient 9	M	19	1993	Undifferentiated carcinoma (EBER unknown)	Nasopharynx T4N1 (AJCC VII edition)	Exclusive RT	Conventional XRT	Neck fibrosis Facial asymmetry Sinonasal synechiae	22 years
Patient 10	F	12	2014	HL	Neck Stage III (Ann Arbor staging)	CT (COPP/ABV × 6); RT above and under diaphragm (total dose 14.4 Gy).	Conventional XRT T and pathological nodes: 62 Gy, elective nodes: 40.2 Gy	-	5 years
Patient 11	M	3	1983	HL	Neck Stage IV (Ann Arbor staging) for bone marrow infiltration	CT + RT	Conventional XRT 20 Gy	-	31 years
Patient 12	M	45	1993	Undifferentiated carcinoma (EBER unknown)	Nasopharynx (stage unknown)	Induction cisplatin + 5-FU followed by exclusive RT	Conventional XRT	-	19 years
Patient 13	M	13	1963	Lymphoma NOS	Supradiaphragmatic lymph nodes	RT above diaphragm	Conventional XRT mantle field (unknown total dose)	-	50 years

Legend: 5-FU, 5-fluorouracil; ABV, adriamycin, bleomycin, vinblastine; AJCC, American Joint Committee on Cancer; CEVAIE, carboplatin, epirubicin, vincristine, actinomycin, ifosfamide, etoposide; COPP, cyclophosphamdie, vincristine, procarbazine, prednisone; CRT, concomitant chemoradiation; CT, chemotherapy; EBER, Epstein–Barr virus-encoded RNA; HL, Hodgkin lymphoma; NOS, not otherwise specified; RT, external beam radiotherapy; Sx, surgery; XRT, photon-based radiotherapy.

**Table 2:** Patient characteristics at clinical presentation of radiation-induced SGC and at follow-up.

Patient	Age at Secondary Diagnosis	Year of SGC Diagnosis	Site and Stage	SGCs Histology	Molecular Analysis	Relationship between SGC Volume and Previous RT Doses and Target Volumes	Treatment of SGCs	Second Course of RT Techniques/RT Dose and Volume	Recurrence/Progression	Toxicity	Last Follow-Up Status	OS Time
Patient 1	29	2016	Parotid gland pT1	Low Grade Polymorphous Adenocarcinoma	N.A.	Within initial treatment volume and marginal to high-dose volume	Sx (R1) → RT	Photon-based VMAT 64 Gy on parotid surgical bed	No	Buccal spasms	Alive NED	36 months
Patient 2	78	2018	Parotid gland cT4 Unresectable disease	Salivary duct carcinoma AR+	PIK3CA: p.E542K exon 9 (6% of DNA) HRAS: p.Q61R exon 3 (13% of DNA) HER-2 negative	Within elective volume and marginal high dose volume	ADT → palliative RT	Photon based VMAT 30 Gy to macroscopic parotid disease (3 Gy per fraction)	Local progression after 2 months	-	Dead ED	13 months
Patient 3	50	2017	Right parotid gland cT4 Unresectable disease	Salivary duct carcinoma AR+	HER-2 negative	Within initial treatment volume	ADT	Not delivered	Local progression and bone metastasis after 6 months	-	Alive ED	24 months
Patient 4	63	2015	Right submandibular gland. Stage pT1N0	Salivary duct carcinoma AR+	HER-2 negative	Not applicable	Surgery for local disease. At diagnosis of metastatic disease chemotherapy (CBDCA/paclitaxel), followed by ADT	Not delivered	Metastatic disease (bone, mediastinal nodes, lung) 12 months after surgery for local disease Best response to systemic treatments: SD to chemotherapy, PR to ADT	-	Alive ED	48 months
Patient 5	68	2016	Parotid pT3R2 (RP nodes radiologically positive)	Carcinoma ex pleomorphic adenoma AR−	NR4A3 negative	Marginal to target volume	Sx → RT	Photon based VMAT-SIB in 33 fractions 69.96 Gy on macroscopic disease+ 59.4 Gy RP nodes and I-II neck levels	No	Fibrosis Trismus Facial nerve disorder	Alive NED	38 months
Patient 6	27	2015	Left parotid pT3 R2	Low grade mucoepidermoid carcinoma	N.A.	Marginal to target volume	Sx → RT	Photon based VMAT-SEQ in 35fractions 70 Gy on residual disease 50 Gy on surgical bed	No	Trismus Facial nerve disorder	Alive NED	48 months
Patient 7	42	2012	Submandibular cT4 (ipsilateral mandibular nerve involvement) Unresectable disease	Adenocarcinoma NOS AR+	N.A.	Marginal to target volume	RT+ADT	Photon based VMAT-SEQ in 30fractions	Brain and bone Progression after 30 months	-	Dead ED	36 months
Patient 8	50	2019	Left submandibular gland pT4R2pN1ECS^+^ Right submandibular gland pT4R2pN0	Adenoid cystic carcinoma (solid variant) Basal cell adenocarcinoma	MYB negative (ACC)	Marginal to high-risk target volume, within elective volume Marginal to high-risk target volume	Sx	Not delivered	Stable disease at both sides	-	Alive ED	4 months
Patient 9	41	2016	Minor salivary gland (Oropharynx)pT2R1N2bECS^+^ M1(bone)	Adenocarcinoma NOS AR+	TP53: p.V216M exon 6 (13% of DNA) HER-2 negative	Not applicable	Sx → ADT	Not delivered	Bone lung brain progression after 25 months from surgery		Dead ED	39 months
Patient 10	17	2019	Parotid pT2 R2	Low Grade Mucoepidermoid carcinoma	N.A.	Marginal to high-risk target volume, within elective volume	Sx → RT	CIRT 6 Gy > Proton therapy (59.4 Gy to parotid bed, 50.4 Gy to perineural path)	No	Trismus Xerostomia Facial and trigeminal nerve disorder	Alive NED	11 months
Patient 11	34 (23 thyroid cancer)	December, 2013 *2005: thiroidectomy and RAI for papillary ca	Parotid pT3R1	ACC	NGS: no mutations ALK and ROS1 negative	Not applicable	Sx → RT	CIRT 68.8 Gy(RBE) (4.3 Gy(RBE) × 16 over 4 weeks)	Lung metastases 2 years after surgery for ACC Currently active surveillance of lung metastases	Trismus Xerostomia Facial and trigeminal nerve disorder	Alive ED	72 months
Patient 12	64	2012	Left parotid M1 (lung)	Salivary duct carcinoma AR+	NGS: BRCA2: p.A336T exon 10 (50% of DNA)	Not applicable	Chemotherapy	Not delivered	Bone, lung and brain progression	-	Alive ED	65 months
Patient 13	63	2013	Right submandibular gland pT4a pN2b	Adenocarcinoma NOS AR+	NGS: no mutations	Not applicable	Sx	Not delivered	Loco-regional, lung and pleural progression	-	Dead ED	74 months

Legend: ACC, adenoid cystic carcinoma; ADT, androgen deprivation therapy [bicalutamide + LHRH analogue]; AR, Androgen receptor; AR+, Androgen receptor overexpression; CBDCA, carboplatin; CIRT, carbon ion radiation therapy; ED, evidence of disease; LHRH, luteinizing hormone releasing hormone; N.A., not assessed; NED, no evidence of disease; NGS, next generation sequencing; PR, partial response; RAI, radioactive iodine; RT, radiation therapy; RP, retropharyngeal node; S, surgery; SD, stable disease; SGC, salivary gland cancer; R1, microscopic residual tumor after surgery; R2, macroscopic residual tumor after surgery; ECS+, extracapsular spread.
